# Supplementary material for: Evolutionary Origin, Genetic Recombination, and Phylogeography of Porcine Kobuvirus
Source: Viruses. 2023 Jan 14;15(1):240. doi: 10.3390/v15010240 (PMC9867129; doi:10.3390/v15010240)
Supplement: Supplementary file 1 [file viruses-15-00240-s001.zip › Supplementary Tables.pdf]

Table S1 Referenced PCV3 strains used in phylogenetic analysis

No.	GenBank Accession No.	Data (Year/Month/Day)	Region	Host
1	AH32	2016.07.13	China	Porcine
2	AH42	2016.07.13	China	Porcine
3	AH49	2016.07.13	China	Porcine
4	AH75	2016.07.13	China	Porcine
5	JN630514	2010.12.15	China	Porcine
6	JQ692069	2011.06.15	China	Porcine
7	JX401523	2012.04.15	China	Porcine
8	KC204684	2011.06.30	China	Porcine
9	KC414936	2012.08.15	China	Porcine
10	KC424638	2012.08.15	China	Porcine
11	KF539763	2012.06.15	China	Porcine
12	KF695124	2008.12.05	China	Porcine
13	KJ452348	2012.06.30	China	Porcine
14	KM051987	2014.03.15	China	Porcine
15	KP144318	2013.06.30	China	Porcine
16	KP260507	2014.12.10	China	Porcine
17	KY234499	2015.06.30	China	Porcine
18	KY234500	2015.06.30	China	Porcine
19	MG800803	2015.09.15	China	Porcine
20	MG800804	2015.09.15	China	Porcine
21	MG800805	2015.09.15	China	Porcine
22	MG800806	2015.09.15	China	Porcine
23	MG800807	2015.09.15	China	Porcine
24	MT125683	2013.12.15	China	Porcine
25	MT125684	2013.12.15	China	Porcine
26	MT125685	2018.09.09	China	Porcine
27	NC016769	2010.12.15	China	Porcine
28	NC027054	2014.12.10	Spain	Porcine
29	MK962321	2017.07.04	Spain	Porcine
30	MK962322	2017.03.16	Spain	Porcine
31	MK962324	2017.09.18	Spain	Porcine
32	MK962327	2017.05.24	Spain	Porcine
33	MK962328	2017.06.01	Spain	Porcine
34	MK962329	2017.11.01	Spain	Porcine
35	MK962330	2017.09.22	Spain	Porcine
36	MK962331	2017.05.30	Spain	Porcine
37	MK962332	2017.07.14	Spain	Porcine
38	MK962333	2017.11.02	Spain	Porcine
39	MK962334	2017.02.14	Spain	Porcine
40	MK962335	2017.01.25	Spain	Porcine
41	MK962336	2017.01.25	Spain	Porcine
42	LT898428	2014.02.14	Germany	Porcine

43	JX177612	2011.04.15	Hungary	Porcine
44	MN807751	2013.01.15	Hungary	Porcine
45	LC210599	2014.01.08	Japan	Porcine
46	LC210600	2014.01.08	Japan	Porcine
47	LC210601	2014.01.08	Japan	Porcine
48	LC210602	2014.01.08	Japan	Porcine
49	LC210603	2014.01.08	Japan	Porcine
50	LC210604	2014.01.09	Japan	Porcine
51	LC210605	2014.01.10	Japan	Porcine
52	LC210606	2014.01.10	Japan	Porcine
53	LC210607	2014.01.10	Japan	Porcine
54	LC210608	2014.01.14	Japan	Porcine
55	LC210609	2014.01.23	Japan	Porcine
56	LC210610	2014.01.29	Japan	Porcine
57	LC210611	2014.02.06	Japan	Porcine
58	LC210612	2015.12.06	Japan	Porcine
59	LC210613	2016.01.20	Japan	Porcine
60	LC210614	2015.11.06	Japan	Porcine
61	LC210615	2015.11.06	Japan	Porcine
62	LC210616	2015.11.05	Japan	Porcine
63	LC210617	2015.11.04	Japan	Porcine
64	LC210619	2015.11.09	Japan	Porcine
65	LC210620	2015.11.27	Japan	Porcine
66	LC210621	2014.04.13	Japan	Porcine
67	LC210622	2014.12.14	Japan	Porcine
68	MT211964	2018.01.18	Mexico	Porcine
69	MZ334483	2018.06.30	Netherland	Porcine
70	KM977675	2011.06.15	America	Porcine
71	MF506730	2011.04.13	America	Porcine
72	OM105002	2021.03.15	South Africa	Porcine
73	MT766372	2018.08.12	China	Sewage
74	MW296158	2019.01.01	China	Sheep
75	GU245693	2009.06.30	Hungary	Sheep
76	NC026314	2011.01.15	Germany	Human
77	AB010145	1998.01.07	Japan	Human
78	AB040749	2000.03.27	Japan	Human
79	JX564249	2010.06.30	China	Human
80	GQ927711	2004.06.30	Germany	Human
81	AY747174	2004.09.10	Germany	Human
82	MF947441	2014.05.19	United Kingdom	Human
83	MK372821	2015.02.02	Pakistan	Human
84	MK372823	2016.02.09	Pakistan	Human
85	FJ890523	2008.06.30	China	Human
86	GQ927712	2004.06.30	Germany	Human

87	GQ927704	2004.06.30	Germany	Human
88	MG200054	2008.01.15	Australia	Human
89	GQ927705	2004.06.30	Germany	Human
90	GQ927706	2004.06.30	Germany	Human
91	DQ028632	2005.05.04	Germany	Human
92	MF947442	2014.07.16	United Kingdom	Human
93	MF598159	2017.06.30	China	Feline
94	KJ958930	2012.06.30	South Korea	Feline
95	MK671315	2018.06.30	China	Feline
96	MK671314	2018.06.30	China	Feline
97	KM091960	2013.03.15	Italy	Feline
98	KF831027	2011.06.30	South Korea	Feline
99	MN337880	2016.04.13	Germany	Canine
100	MN449341	2019.04.15	China	Canine
101	MH747478	2016.06.30	Brazil	Canine
102	KF924623	2012.06.15	South Korea	Canine
103	NC034971	2015.09.15	China	Canine
104	KC161964	2008.06.30	United Kingdom	Canine
105	JQ911763	2011.06.30	China	Canine
106	JN088541	2010.06.30	America	Canine
107	MW605074	2021.12.12	America	Bovine
108	MN336260	2019.06.13	America	Bovine
109	KY407744	2014.06.30	Egypt	Bovine
110	ON075056	2021.06.30	China	Bovine
111	ON075055	2021.06.30	China	Bovine
112	ON075054	2021.06.30	China	Bovine
113	ON075053	2021.06.30	China	Bovine
114	ON075052	2021.06.30	China	Bovine
115	ON075051	2021.06.30	China	Bovine
116	ON075050	2021.06.30	China	Bovine
117	MZ603734	2021.07.20	China	Bovine
118	KJ641686	2010.10.15	China	Bat
119	KJ641691	2012.12.15	China	Bat
