# Supplementary figures and images for: Evolutionary Origin, Genetic Recombination, and Phylogeography of Porcine Kobuvirus
Source: Viruses. 2023 Jan 14;15(1):240. doi: 10.3390/v15010240 (PMC9867129; doi:10.3390/v15010240)

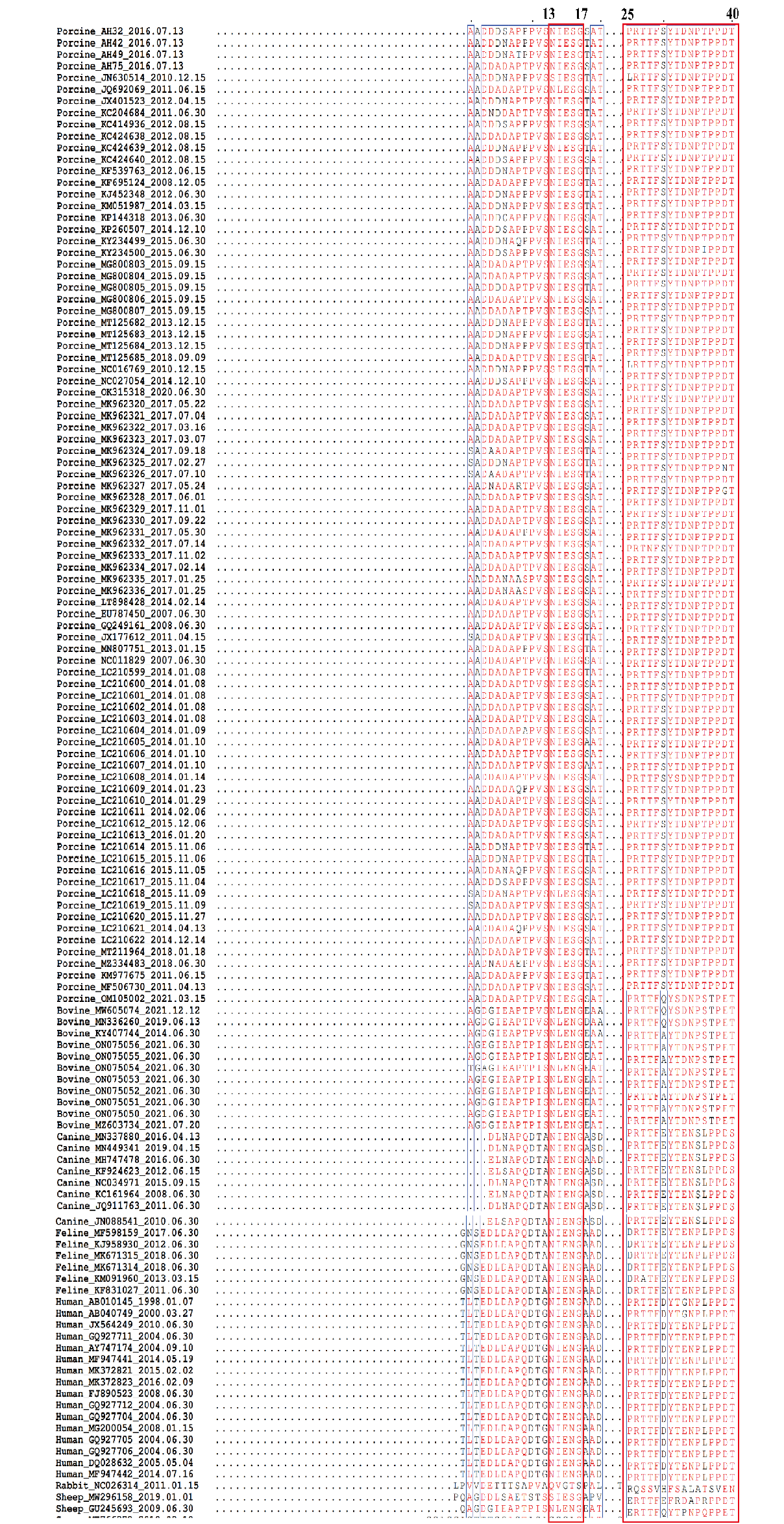

Supplement: Supplementary file 1 [file viruses-15-00240-s001.zip › Supplementary Figures/Figure S1.tif]

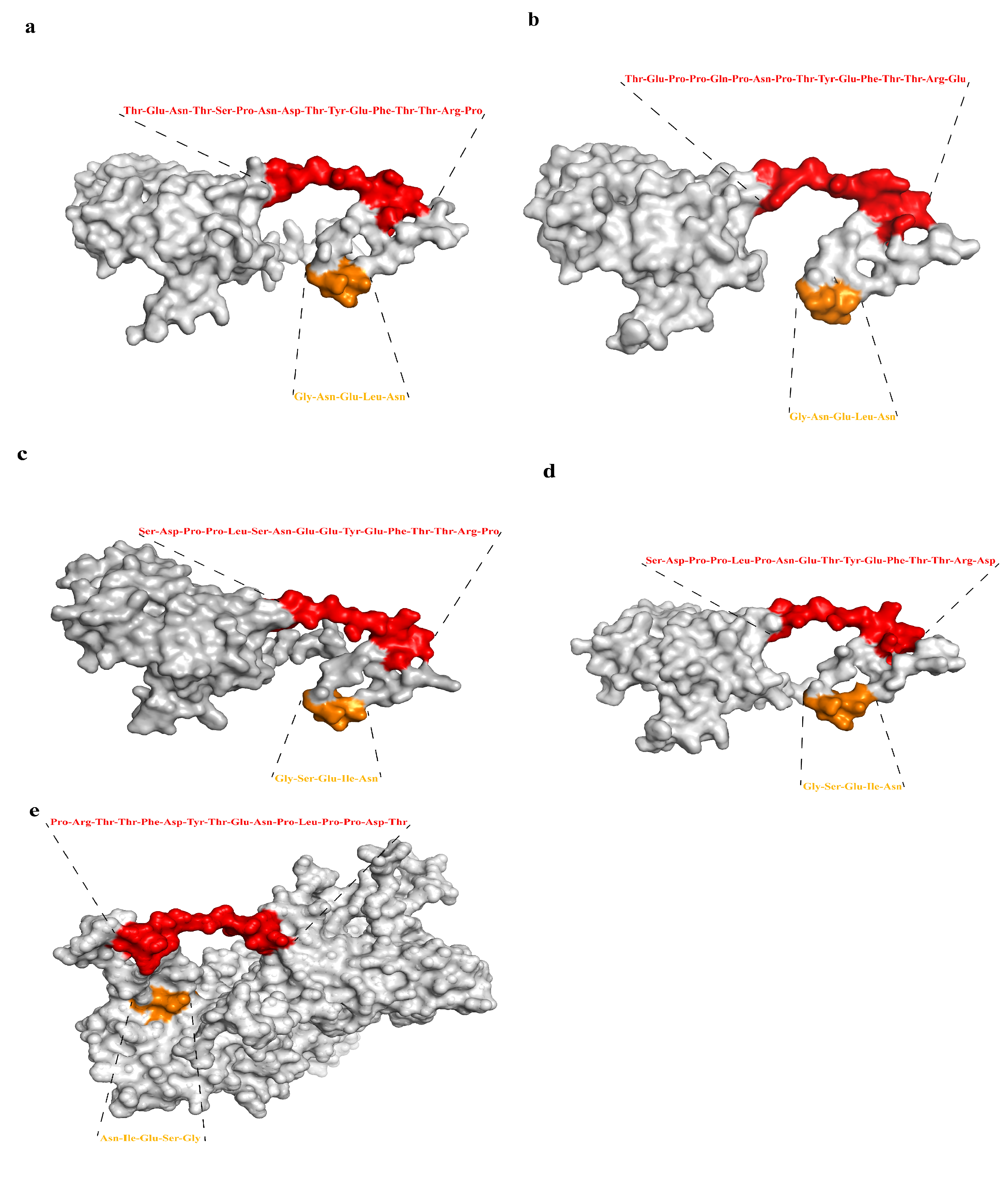

Supplement: Supplementary file 1 [file viruses-15-00240-s001.zip › Supplementary Figures/Figure S2.tif]

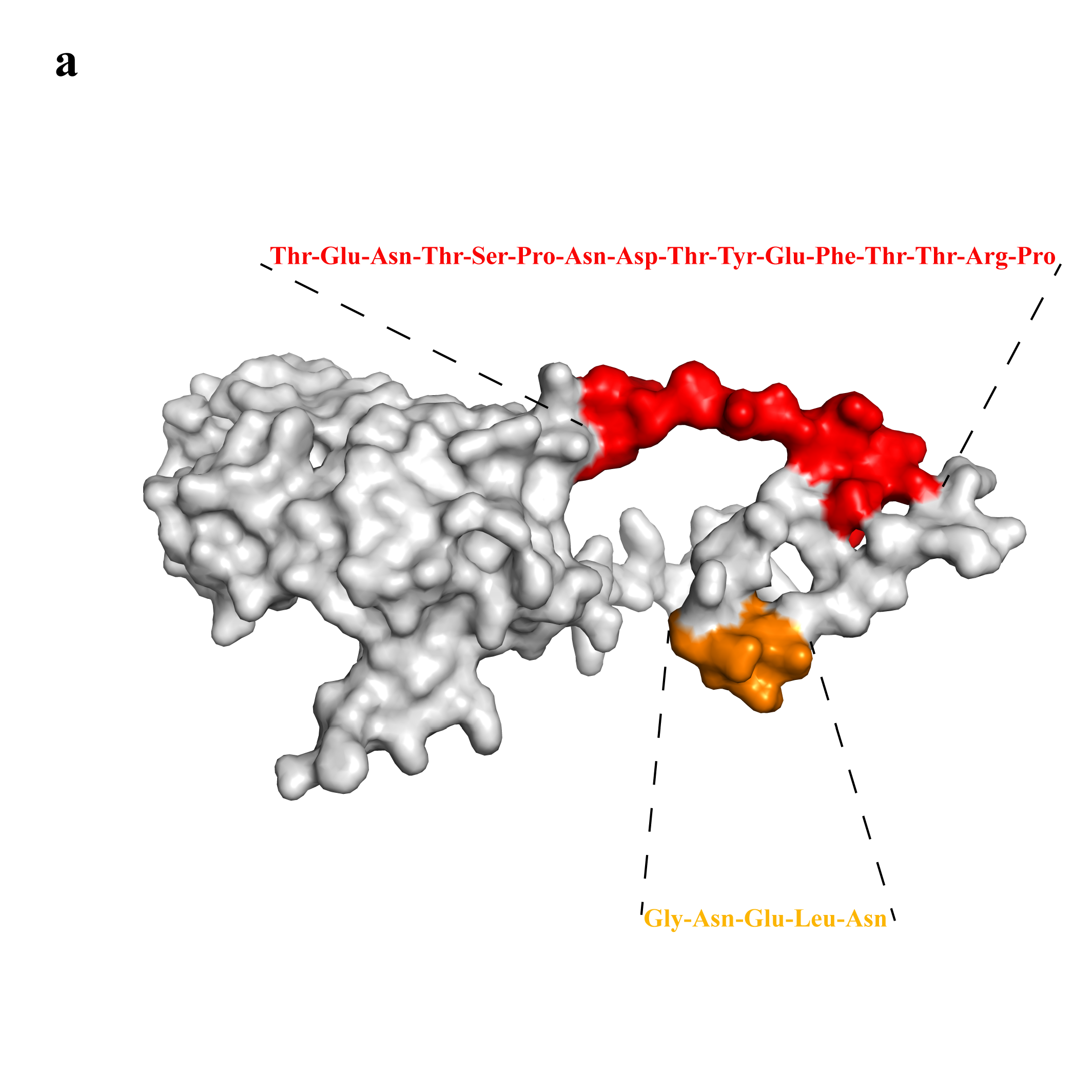

Supplement: Supplementary file 1 [file viruses-15-00240-s001.zip › Supplementary Figures/Figure S2a.tif]

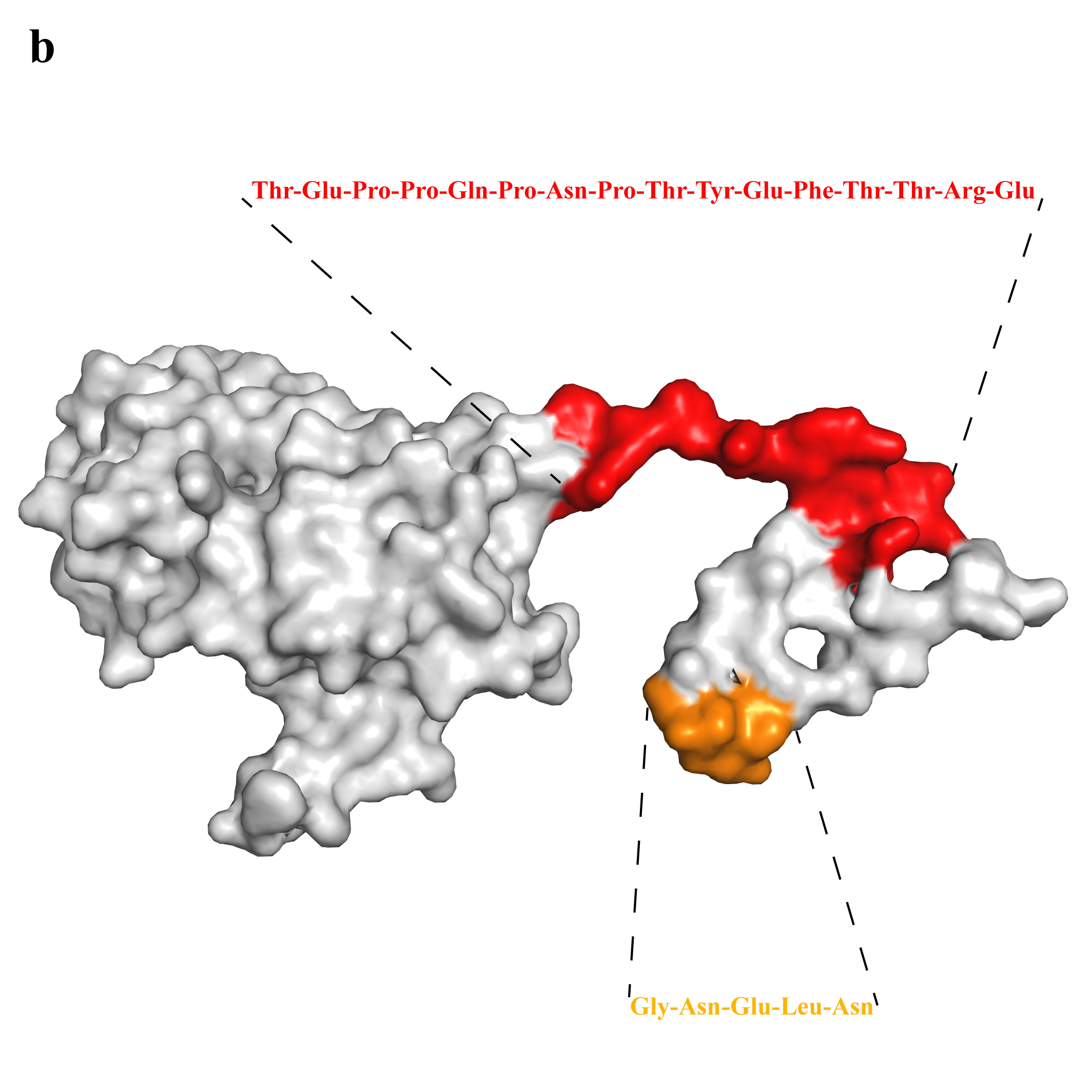

Supplement: Supplementary file 1 [file viruses-15-00240-s001.zip › Supplementary Figures/Figure S2b.tif]

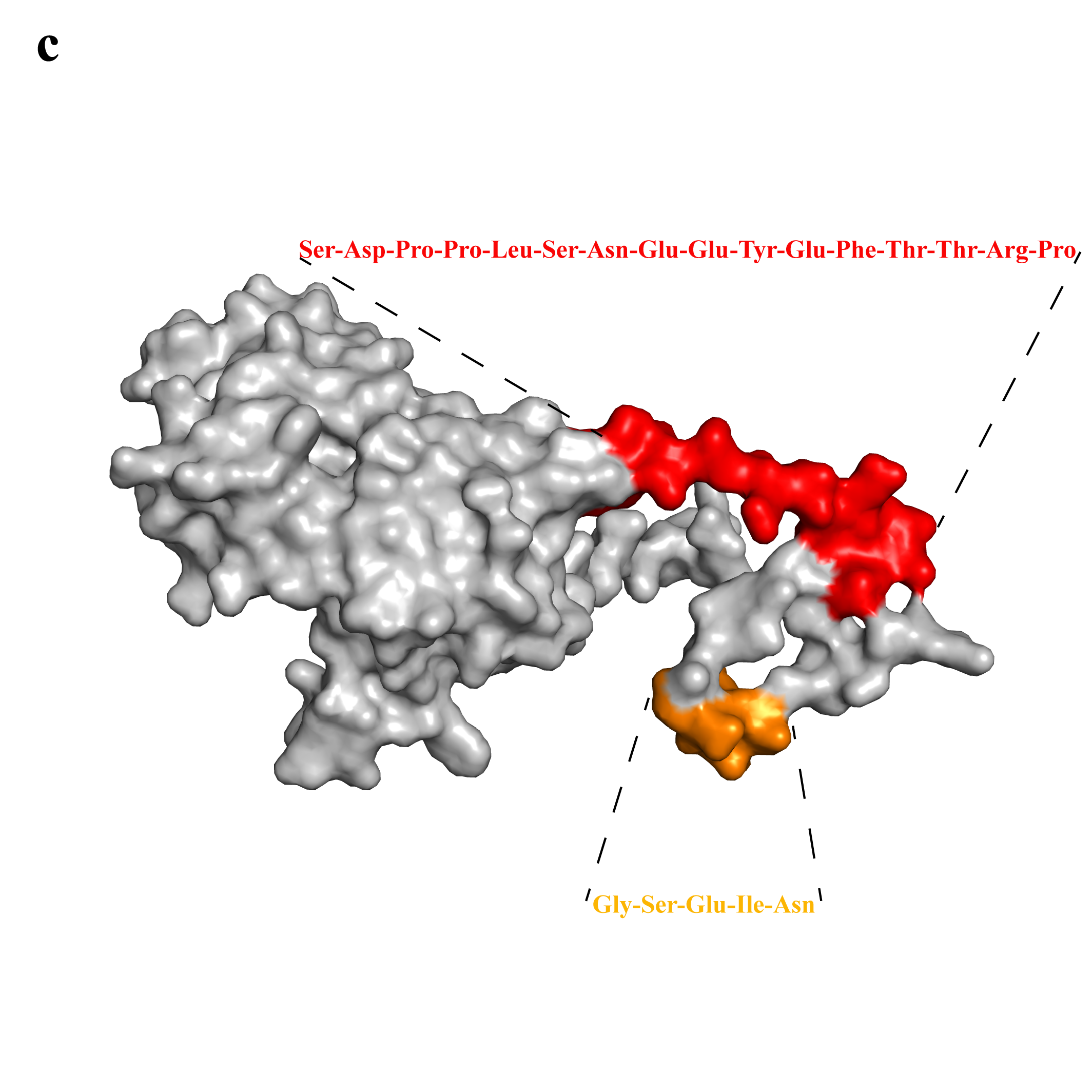

Supplement: Supplementary file 1 [file viruses-15-00240-s001.zip › Supplementary Figures/Figure S2c.tif]

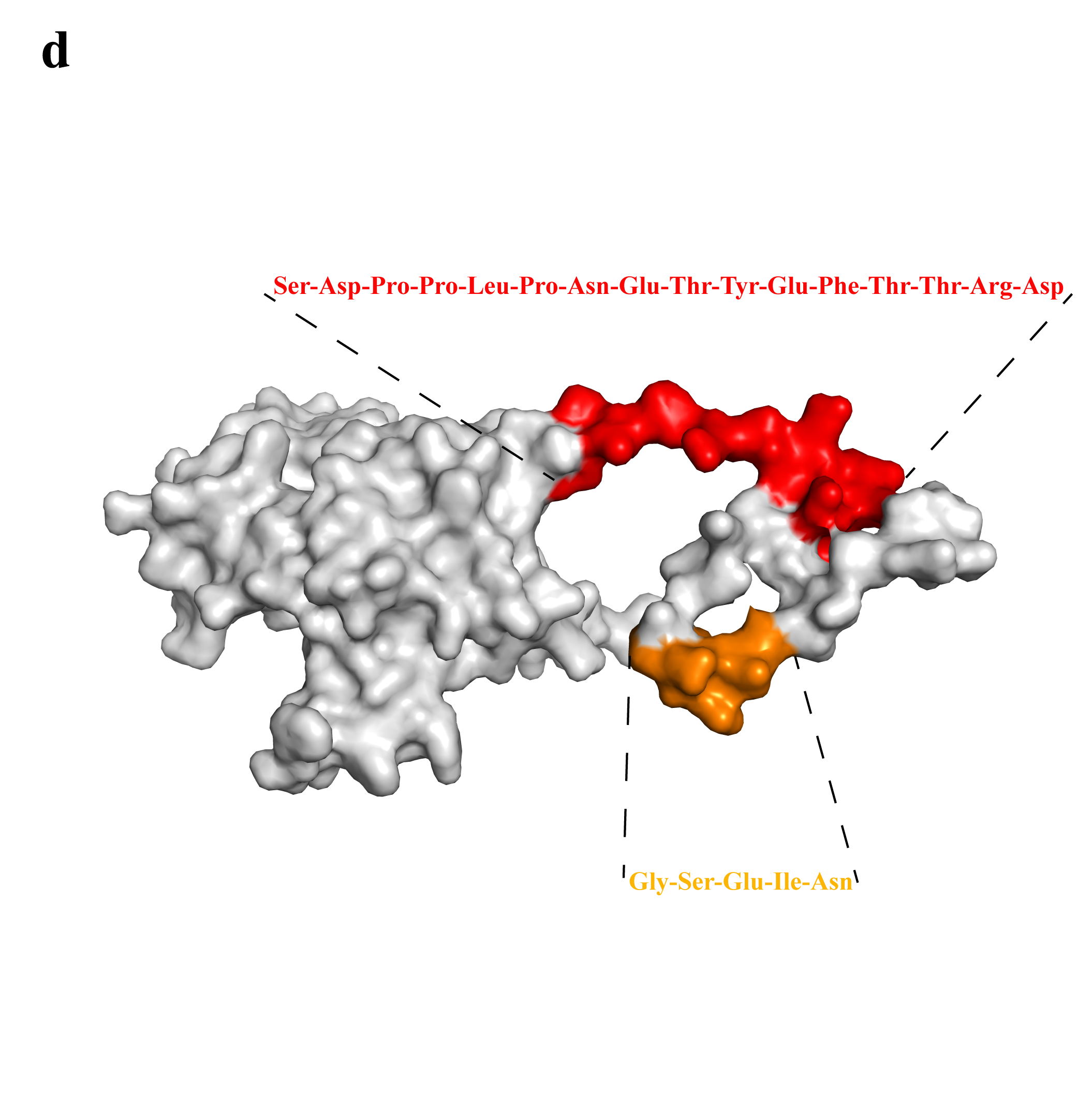

Supplement: Supplementary file 1 [file viruses-15-00240-s001.zip › Supplementary Figures/Figure S2d.tif]

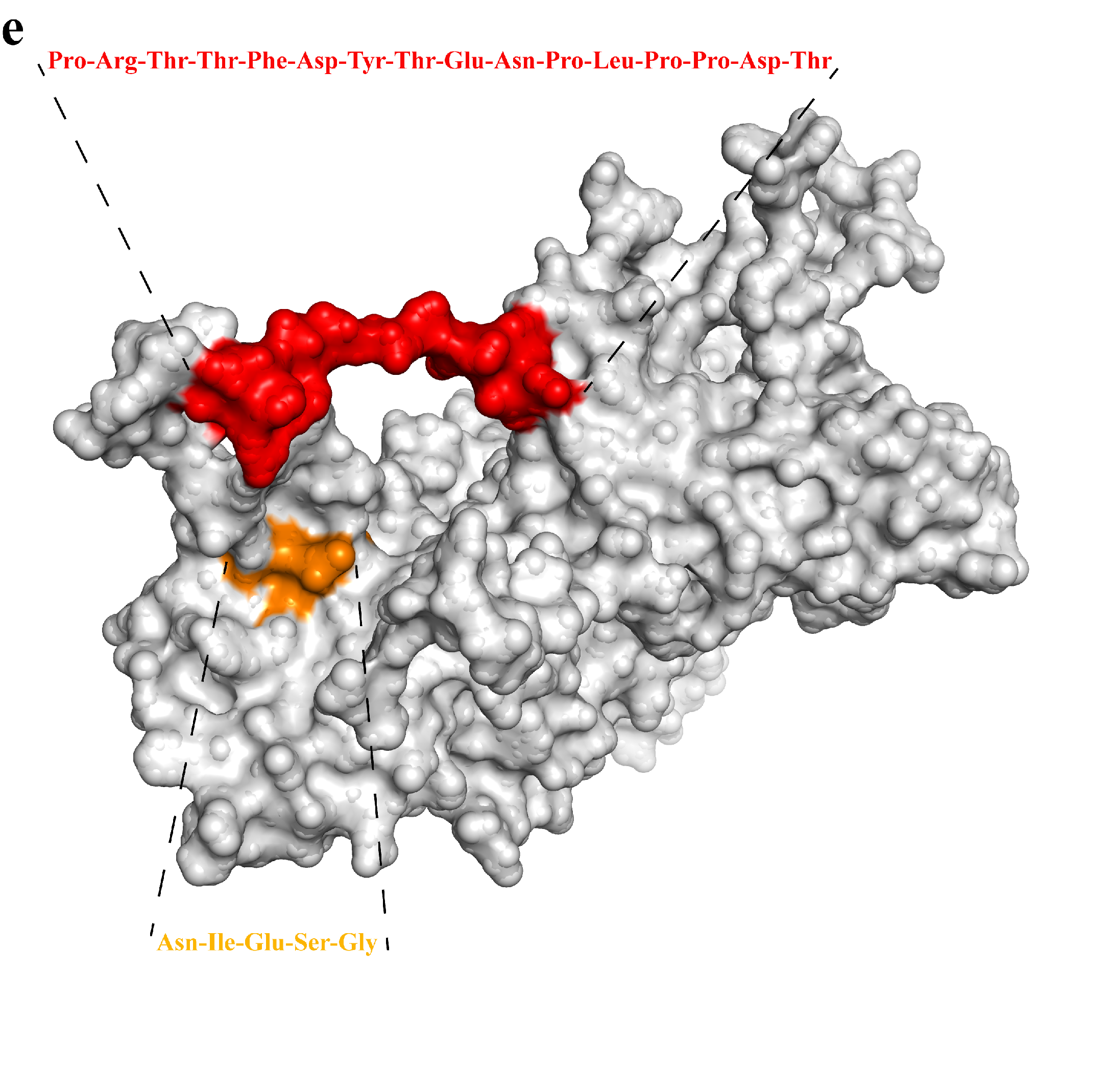

Supplement: Supplementary file 1 [file viruses-15-00240-s001.zip › Supplementary Figures/Figure S2e.tif]

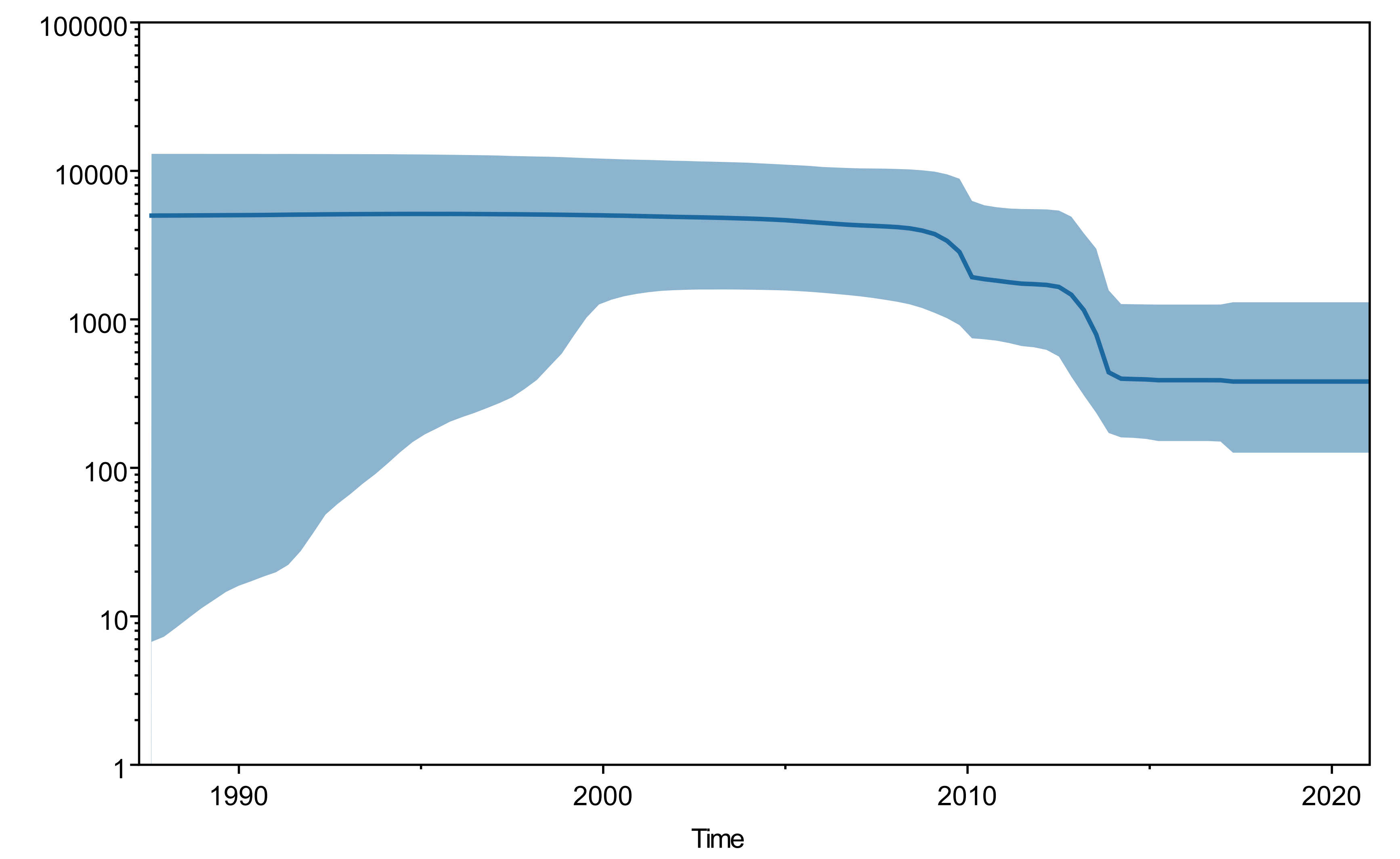

Supplement: Supplementary file 1 [file viruses-15-00240-s001.zip › Supplementary Figures/Figure S3.tif]
